# Surface Modification and Damage of MeV-Energy Heavy Ion Irradiation on Gold Nanowires

**DOI:** 10.3390/nano7050108

**Published:** 2017-05-15

**Authors:** Yaxiong Cheng, Huijun Yao, Jinglai Duan, Lijun Xu, Pengfei Zhai, Shuangbao Lyu, Yonghui Chen, Khan Maaz, Dan Mo, Youmei Sun, Jie Liu

**Affiliations:** 1 Institute of Modern Physics, Chinese Academy of Sciences (CAS), Lanzhou 730000, China; chengyaxiong@impcas.ac.cn (Y.C.); j.duan@impcas.ac.cn (J.D.); xulj@impcas.ac.cn (L.X.); zhaipengfei@impcas.ac.cn (P.Z.); lvshuangbao@impcas.ac.cn (S.L.); yhchen@impcas.ac.cn (Y.C.); maaz@impacs.ac.cn (K.M.); modan@impcas.ac.cn (D.M.); ymsun@impcas.ac.cn (Y.S.); 2 School of Physical Sciences, University of Chinese Academy of Sciences (UCAS), Beijing 100049, China; 3 Nanomaterials Research Group, Physics Division, PINSTECH, Nilore, Islamabad 45650, Pakistan

**Keywords:** gold nanowires, ion irradiation, surface modification, stacking fault tetrahedrons

## Abstract

Gold nanowires with diameters ranging from 20 to 90 nm were fabricated by the electrochemical deposition technique in etched ion track polycarbonate templates and were then irradiated by Xe and Kr ions with the energy in MeV range. The surface modification of nanowires was studied by scanning electron microscopy (SEM) and transmission electron microscopy (TEM) characterizations. Different craters with and without protrusion on the gold nanowires were analyzed, and the two corresponding formation mechanisms, i.e., plastic flow and micro-explosion, were investigated. In addition, the sputtered gold nanoparticles caused by ion irradiation were studied and it was confirmed that the surface damage produced in gold nanowires was increased as the diameter of the nanowires decreased. It was also found that heavy ion irradiation can also create stacking fault tetrahedrons (SFTs) in gold nanowires and three different SFTs were confirmed in irradiated nanowires. A statistical analysis of the size distribution of SFTs in gold nanowires proved that the average size distribution of SFT was positively related to the nuclear stopping power of incident ions, i.e., the higher nuclear stopping power of incident ions could generate SFT with a larger average size in gold nanowires.

## 1. Introduction

Nanomaterials have received immense interest in recent years due to their vast applications in modern technology. It has been shown that the properties of nanomaterials are quite different from their bulk counterparts, due to their enhanced surface effects, high interface-to-volume ratio, and small size. The interaction between energetic ions and solids has been extensively studied for many years. A number of excellent works have been carried out, which have revealed the transport process of energetic particles in solids. New models such as the cascade collision, displacement spike, and thermal spike have been introduced, and these can be used to explain the damages produced in solids as a result of heavy ion irradiation [1,2]. Based on molecular dynamics simulations, there is a good understanding on the microscopic processes which occur during energetic particle irradiation [3,4]. Energetic heavy ion irradiation usually leads to the damages on both the surface and bulk of the heavy metals, resulting in craters and hillocks on the surface of the materials [5], and stacking fault tetrahedrons (SFTs) in the bulk [6,7]. Irradiation of gold film with 50–400 keV Xe ions provides evidence that the plastic flow process can generate craters and protrusions on the gold film [8,9,10]. Recently, a study on the MeV self-ion irradiation of gold nanoislands carried out by P.V. Satyam et al. indicated that thermal spike confinement potentially generates craters within the gold nanoisland [11]. K. Nordlund and F. Gao have studied irradiation-induced SFTs below the vacancy migration temperature in metals through molecular dynamics simulation [12], which indicates that SFTs can be directly created in the energetic collision cascade. The study of irradiation-induced SFTs in face-centered cubic (FCC) metal by Schaublin et al. clarifies the structure of SFTs in the metal [7].

Recently, the irradiation effects in nanomaterials by energetic heavy ions have become a hot topic in nanoscience, with both beneficial and detrimental effects appearing in the materials. Energetic particle irradiation can serve as a tool to modify material properties at a nanoscale, such as the improvement of electrical properties of semiconductor nanowires (NWs) by ion implantation [13], which cannot be achieved using traditional techniques [14]. The ions with energies between 500 and 2000 eV possess unique and attractive capabilities which can contribute to the topography engineering of the surface at nanometer and micron length scales [15], and the lower energy plasmas with an energy of approximately hundreds of eV are capable of controllably roughing polymeric and silicon surfaces, or forming organized nanostructures on such surfaces through the plasma-wall interaction [16]. Research on the negative effects due to ion irradiation is also important in the case of nano-electronic devices working in the irradiation environment. Although irradiation-induced damages have been studied for a long time, very limited research has been carried out on the damages induced in nanowires as a result of the heavy ions with an energy in the MeV range.

In this paper, we report a study of the irradiation effects on a single crystalline gold nanowire, which are fabricated electrochemically in the etched ion track templates. After irradiation, the variation of the morphology and crystal structure of the gold nanowires are characterized and evaluated by using scanning electron microscopy (SEM) (FEI, Hillsboro, OR, USA) and high-resolution transmission electron microscopy (HRTEM) (FEI, Hillsboro, OR, USA).

## 2. Results and Discussion

**Figure 1:**
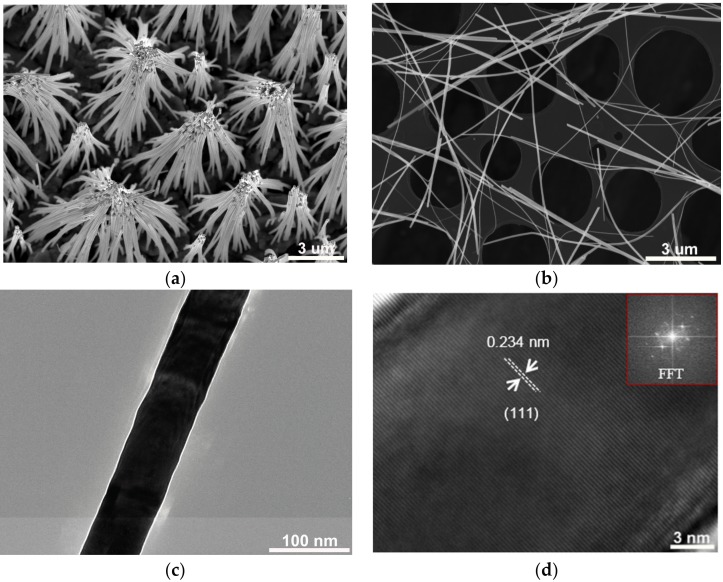
Scanning electron microscopy (SEM) images of gold nanowires (NWs) (**a**) Prepared on Au/Cu substrate; (**b**) Transferred to transmission electron microscopy (TEM) grid; (**c**) TEM image of 70 nm gold NW; (**d**) High resolution TEM image of gold NW which indicates growing direction of [111]. The diameter is 30 nm and the inset is the fast Fourier transform (FFT) image of this area.

The as-prepared gold NWs on the Au/Cu substrate were first characterized by SEM after dissolving the polycarbonate (PC) template, as shown in Figure 1a. The NWs of tens of micrometers in length can be observed on the substrate, which are aggregated with each other. This aggregation phenomenon normally occurs during the sample preparation, which can be ascribed to the high aspect ratio of NWs and the surface tension of the solvent drops while drying the sample [17,18]. Gold NWs with diameters from 20 to 90 nm were fabricated to study the size dependence of irradiation damage induced by the heavy ions. To produce the same irradiation fluence for different nanowires, the NWs with different diameters were mixed together and transferred to the TEM (FEI, Hillsboro, OR, USA) grid, as shown in Figure 1b. In this way, the gold NWs with different diameters were presented on the same TEM grid and underwent the same ion irradiation. The gold nanowire with a diameter of 70 nm is shown in Figure 1c, presenting a smooth surface and excellent cylindrical shape. The crystal structure of the prepared nanowires was also investigated by HRTEM. Figure 1d exhibits the single crystalline structure and [111] growing direction of a 30 nm gold NW. More structural information on the gold NWs with various diameters and preparation conditions can be found in our previous study [19].

### 2.1. Surface Modification

**Figure 2:**
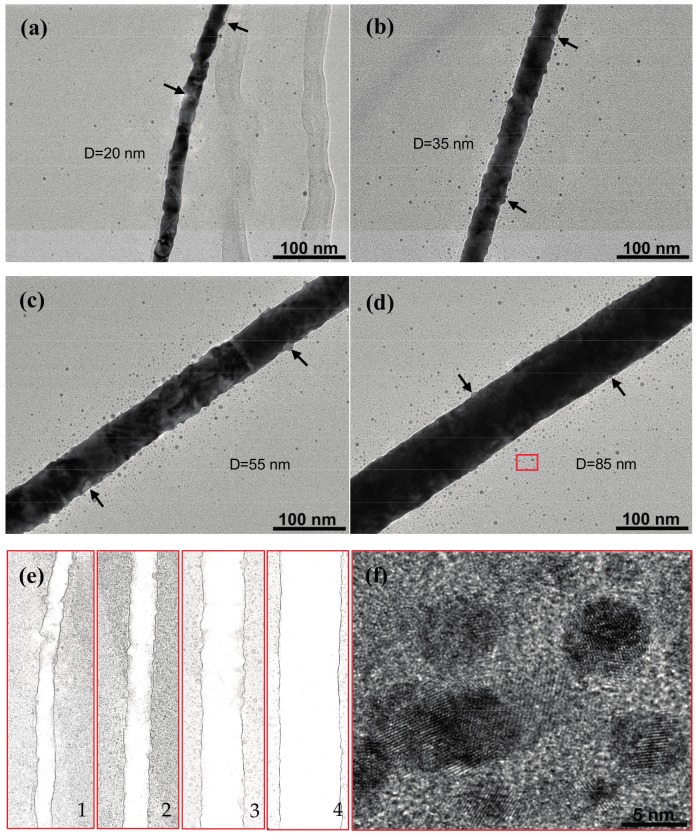
TEM images of gold NWs after ion irradiation with 1.4 MeV Xe ions, at a fluence of 1 × 10^14^ ions cm^−2^. The diameters of these NWs are (**a**) 20 nm, (**b**) 35 nm, (**c**) 55 nm, and (**d**) 85 nm, respectively. Black arrows point out products on NWs. (**e**) 1, 2, 3, and 4 show the contour of irradiated gold NWs in (**a**–**d**), separately. (**f**) High-resolution transmission electron microscopy (HRTEM) image of sputtered NPs of rectangular region in (**d**).

To investigate the morphology changes caused by the heavy ion impact, the irradiated gold nanowires were characterized by TEM. The gold NWs with four different diameters, including 20, 35, 55, and 85 nm, were investigated after heavy ion irradiation, to determine the size effect. In this case, the gold NWs were irradiated with 1.4 MeV Xe ions and the irradiation fluence was maintained at 1 × 10^14^ ions cm^−2^. As shown in Figure 2a–d, a large number of protrusions and craters on the surface of the gold nanowires, as indicated by the black arrows, can be clearly seen. In order to observe the morphology changes more clearly, the image contrast was adjusted by Adobe Photoshop (CC-2015) (Adobe Systems Incorporated, San Jose, CA, USA). For a good comparison, the same magnification and scale bar were chosen in Figure 2a–d. In these images, it is seen that the gold nanowire surface is roughened by ion irradiation and that new microstructures are produced on the surface after ion bombardment, some of which are pointed out by the arrows. Similar phenomena have also been observed in other works [9,10], which confirm that the irradiation products, like craters or hillocks (in the form of a protrusion or adsorbed particle), can be found on gold foils after irradiation with heavy ion beam. In the case of gold nanowires, the products can be generated all around the nanowire due to its quasi-one-dimensional structure features, in contrast with the gold foils. As can be seen in Figure 2a, the ion impacts produce considerable craters and protrusions on the edge of the nanowires, resulting in jagged contours in the case of the 20 nm gold NWs. Here, the protrusion size is measured as 15.4 nm in width and 6.7 nm in height, and the crater is about 11.5 nm in width and 6 nm in depth, i.e., one third of the nanowire diameter. Huge craters with a depth reaching half of the nanowire diameter were also found in our experiment. These types of craters should be considered not only as surface damage, but also as bulk damage, which probably significantly influence the electronic and mechanical properties of gold NWs. The surface of the gold NWs with diameters of 35 and 55 nm were found to be less modified by the heavy ions (Figure 2b,c), when compared with the 20 nm nanowire. The thicker NW with a diameter of 85 nm also shows craters, protrusions, and adsorbed particles (Figure 2d). However, the main difference is that the thicker NWs are still in intact contours when compared with the thinner ones, after being irradiated at the same condition. As can be seen in the images, the degree of contour change increases with a decreasing nanowire diameter. This phenomenon can be attributed to the small size and surface effect of the nanostructures. Supported by the larger surface-to-volume ratio of the nanowires, a higher fraction of gold atoms resides on the surface, so that more damage processes can occur on the surface of the nanowires. This can result in the enhanced surface damages and high sputtering yields in the case of thin NWs [20]. Figure 2e gives a detailed comparison of the contour changes on irradiated gold NWs (Figure 2a–d).

Heavy ion irradiation can also lead to nanoscale-particle sputtering from the gold nanowire surface. As shown in Figure 2a–d, the sputtered particles around the gold nanowires are collected by the carbon membrane on the TEM grid. Most of the sputtered particles distributed around the parent NWs are in the range of 200 nm as a Gaussian distribution. As the nanowire’s diameter increases, the amount of the sputtered particles increases due to the increased impact of the cross section under the same irradiation conditions. In these images, the sputtered particles are confirmed to be possessing twin or polycrystalline structures. These NPs are considered as the molten gold ejected from a displacement cascade in gold NWs [21]. The variability in the shape of the sputtered NPs may arise from the subsequent heavy ion impact. The HRTEM micrograph of gold NPs shown in Figure 2f is the rectangular area in Figure 2d with a higher magnification, which confirms the presence of the sputtered gold nanoparticles.

**Figure 3:**
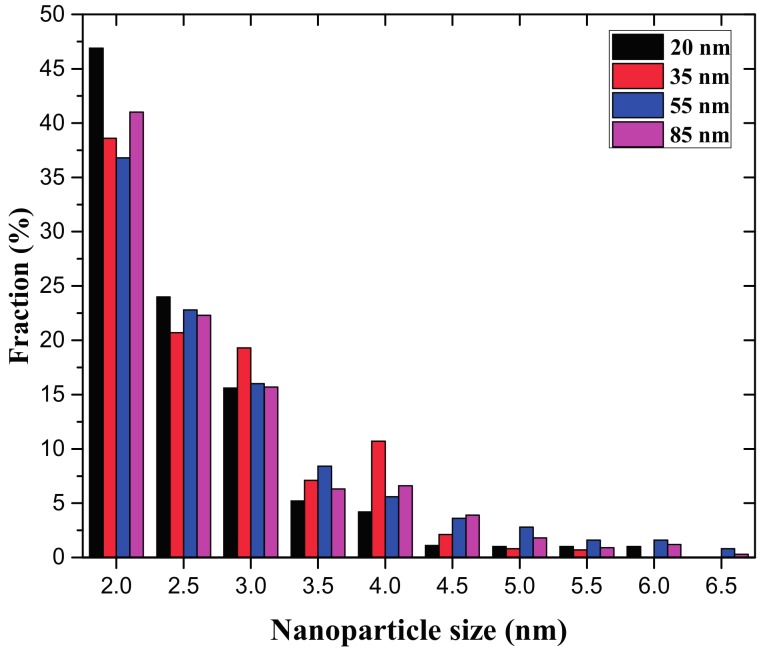
Size distribution of sputtered gold clusters ejected from gold NWs after ion irradiation. The gold NWs with a diameter ranging from 20 to 85 nm were irradiated by 1.4 MeV Xe with the fluence of 1 × 10^14^ ions cm^−2^.

In order to collect information about the size distribution of the sputtered gold particles from four different gold NWs, statistical work was carried out and the corresponding histogram is shown in Figure 3. To make the statistic work easier, only the sputtered gold nanoparticles with a size larger than 2 nm are taken into account in this work. The number of sputtered particles decreases as the particle size increases, and more than 75% of particles display a size smaller than 3 nm in all cases. It was also found that almost no particles were present with sizes larger than 7 nm in the entire field. It can be concluded that the size distribution of the particles sputtered from gold NWs with different diameters exhibits a slight difference, i.e., the nanowires with a larger diameter can sputter bigger particles than the ones with a smaller diameter. This phenomenon may be ascribed to the lower adsorption energy of the thicker NWs, and with a decreasing diameter, the dangling bond of the gold atoms on the NW surface increases, which results in the higher adsorption energy. Therefore, under the heavy ion impact, there are more NPs likely to be sputtered from the nanowire, instead of adhering on its surface.

**Figure 4:**
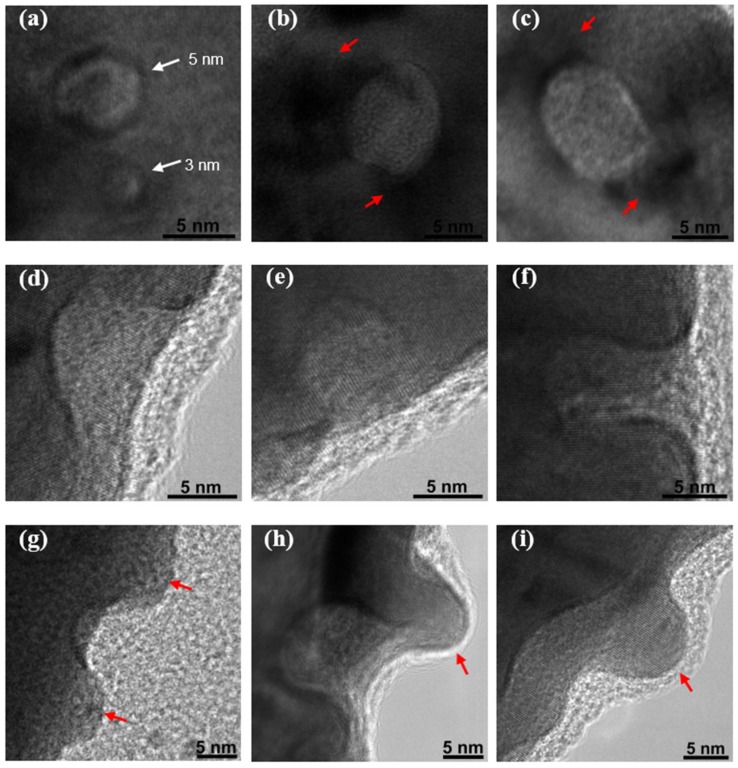
HRTEM images of typical features of a crater on irradiated NWs. (**a**–**c**) Craters on top surface. (**d**–**f**) Craters without protrusion on side surface, and (**g**–**i**) Craters with protrusion nearby. Red arrows point out protrusions on NW.

If we further observe the irradiated gold NWs with HRTEM, more details of the craters produced by the ion impacts can be found, as shown in Figure 4. These craters not only distribute on the top surface, but also on the lateral surface of the NWs. Figure 4a–c shows the craters formed on the top surface. Since the HRTEM images were obtained with an under-focused condition, the bright and dark areas indicate the cavities and protrusions, respectively. In Figure 4a, two craters with diameters of 3 and 5 nm show the clear bright center and dark rim surrounding it. Besides this, in Figure 4b,c, the craters with an ellipse-like shape can also be found on the irradiated NWs, whose size is far beyond that in Figure 4a. These craters have lengths beyond 7 nm in the short axis and 10 nm in the long axis. As for the craters shown in Figure 4b,c, there is an irregular dark region instead of the broad dark rim nearby, which is considered to be the recrystallized matter from the crater that forms the protrusion, as pointed out by the red arrows. This difference may arise from two different formation mechanisms, which are the plastic flow and micro-explosion.

In addition, two kinds of craters can be found on NW’s side part. One is without protrusion, as shown in Figure 4d–f , and the other one is the crater with protrusions nearby, as shown in Figure 4g–i. Various shapes of the formative craters are shown in these micrographs. The crater shown in Figure 4d possesses a V-shape, while those shown in Figure 4e,f possess a U-shape. The crater in Figure 4g shows the feature of a cavity with two protrusions nearby, whereas the craters in Figure 4h represented by a U-like shape are accompanied by one big protrusion. Moreover, the crater shown in Figure 4i displays different structural features, as its size is beyond 25 nm along the axial direction of the NWs. Such a large size indicates that it was formed as a result of more than one heavy ion impact.

**Figure 5:**
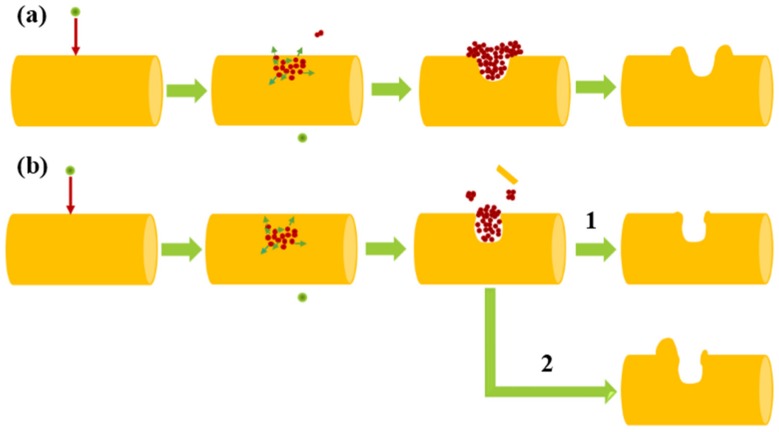
A sketch map of the crater formation mechanism. (**a**) Plastic flow process; (**b**) micro-explosion process, 1 and 2 point out the formation of the crater without and with a protrusion, respectively.

The craters displayed in Figure 4 indicate the frequent transport of matter. The volume of the crater could contain more than 5000 gold atoms. Previous studies indicate that the spike effect induced by heavy ion irradiation can produce large craters on the surface of NWs [10,22]. Two basic processes for the formation of craters are plastic flow and micro-explosion [8,9,23]. A sketch map of the crater formation process by the spike effect is described in Figure 5. The incidence of an MeV heavy ion can generate displacement cascades in gold NWs [2]. As the displacement cascade happens on only several atomic layers beneath the surface, the molten gold in the thermal spike phase is able to expand freely to the surface, and flow to the surface by the surface energy, forming the crater with a dark rim [9,10,24]. This process is shown in Figure 5a, and when the displacement cascade occurs at a deeper level, the molten gold can’t directly move to the surface, forming a confined spike. Due to the high localized pressure, the confined pressure spike can reach up to 10 GPa to push out the solid plug on the NW surface, which causes the molten gold to be explosively ejected out and form protrusions on the surface [9,10]. The ejected gold can adhere to the surface of the NWs when its kinetic energy is less than the adsorption energy of the NWs, while the ejected gold will leave the NW when the kinetic energy is large enough to escape from the NW (named micro-explosion process, in Figure 5b). This ejected molten gold could fall on the NW surface as a protrusion near the crater or as a deposit on the carbon membrane, to form the NPs [21,23].

### 2.2. Stacking Fault Tetrahedron in Bulk

**Figure 6:**
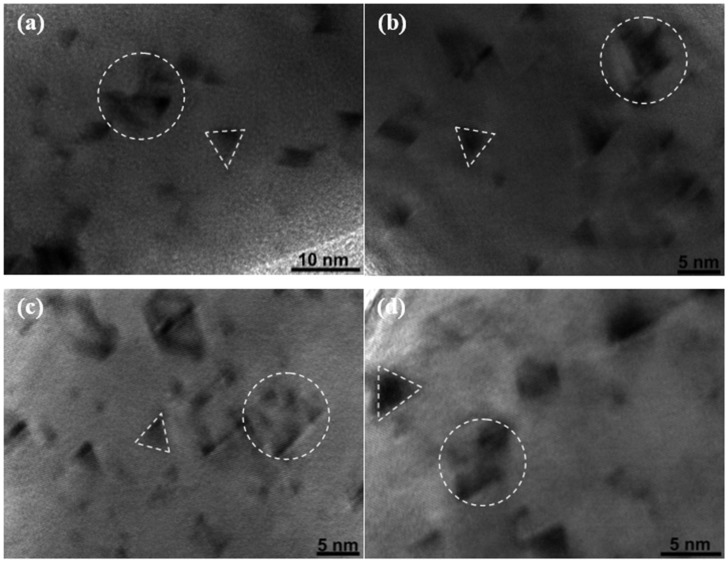
High-resolution TEM micrographs of irradiated gold nanowire. Gold NWs irradiated with (**a**) 1.4 MeV Xe ions, (**b**) 4 MeV Xe, (**c**) 1.4 MeV Kr, and (**d**) 3 MeV Kr. Dashed triangles and circles indicate single stacking fault tetrahedrons (SFTs) and group of intermixed SFTs, respectively.

In this work, the projected range of the heavy ions in gold is far beyond the maximum diameter of gold NWs (90 nm) according to the theory calculations (Structure Reaction Injection Molding (SRIM)-2013) shown in Table 1, and the incident heavy ions can pass through the NWs in a normal irradiation mode. Therefore, the irraidiation impact can not only modify the surface morphology, but also can produce defect clusters inside the NWs. The structure modification in gold NWs was characterized by using bright field HRTEM. Figure 6a–d gives the typical features of the structure induced by Xe and Kr ions with different energies in the MeV range. As can be seen in these images, the ion impacts can produce low contrast defect clusters, which have triangular and polygonal shapes. Most of these structures are considered to be stacking fault tetrahedrons (SFTs). The geometrical shape of these SFTs is a tetrahedron, which is formed by the four vacancy-type stacking faults on {111} planes intersecting along the <110> edges [25]. Such defect clusters can easily be produced in metals with low stacking fault energies [26].

In Figure 6, the projection plane is {110} and the triangular shape is the image of a single SFT projected on the {110} plane. Polygonal shapes indicate a group of intermixed SFTs. These single SFTs and SFT groups (in Figure 6a–d) are pointed out by the dashed triangles and circles, respectively. It has been reported that a vacancy-type stacking fault tetrahedron can be formed directly in a collision cascade [7,12]. Ion irrdiation can generate a displacement spike in gold NWs. The nature of the displacement spike is a hot zone where the atoms are expelled by the displacement collision, and as a result, an atom depletion region is formed [2]. In this case, the formation of an SFT during the irradiation process is strongly related to the displacement spike and it has been simulated that SFT can be directly formed by the collapse of an atom depletion region in the center of the cascade [12]. The molecular dynamics simulation [27] has demonstrated the formation of SFTs in irradiated gold NWs and proved our explanation on SFT formation.

**Figure 7:**
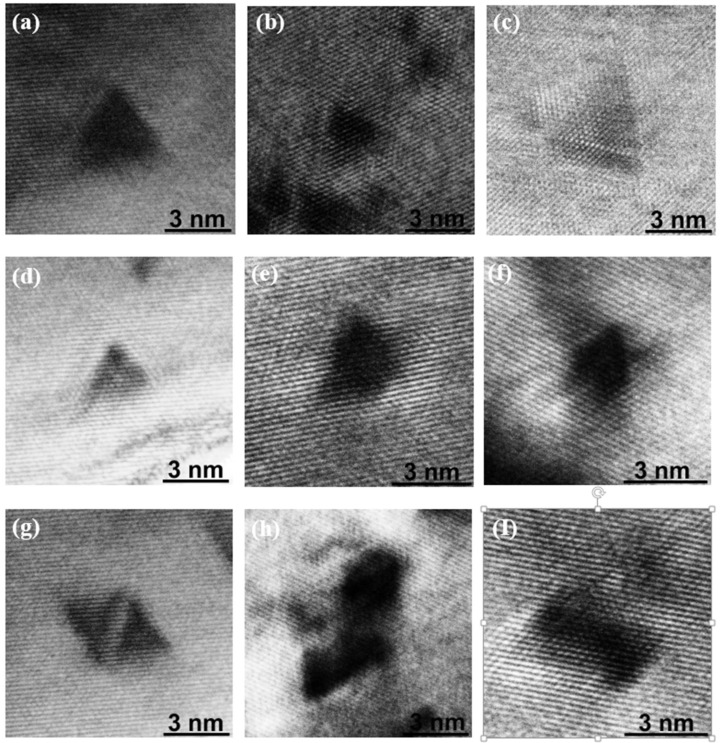
TEM micrographs of SFTs in NWs irradiated by MeV heavy ions. (**a**–**c**) Perfect SFTs, (**d**–**f**) truncated SFTs, and (**g**–**i**) group of intermixed stacking fault tetrahedron (SFT).

Different features of SFTs in these irradiated gold nanowires can be confirmed, as shown in Figure 7. Complete SFTs, truncated SFTs, and SFT groups are presented in Figure 7a–i, respectively. As is illustrated in these images, the complete SFTs present the intact triangle shape and for the truncated SFTs, they are formed as part of an intact triangle being cut away. The SFT groups can be in the form of a regular parallelogram or in irregular shapes. A simulation shows that an SFT group can also arise directly from a single cascade [7]. Most of the SFTs have a side length between 2 nm and 4 nm. However, the largest one can reach up to 7 nm, which is formed in a high energy density collision cascade.

**Figure 8:**
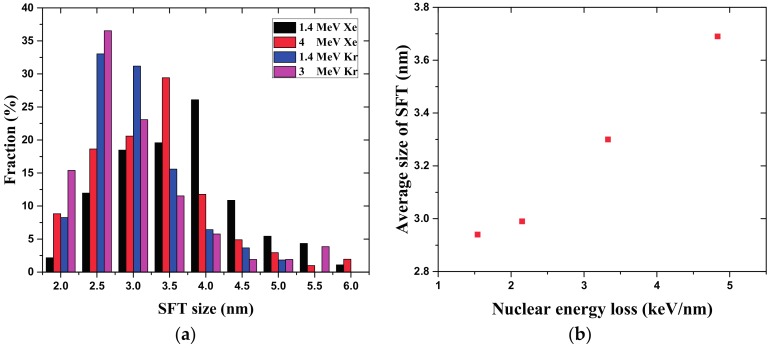
Size distribution of SFTs in gold nanowires. (**a**) The fraction of different SFT; (**b**) The relationship between average size of SFT and nuclear energy loss.

By counting more than 100 SFTs in gold NWs irradiated by Xe and Kr ions with different energies and choosing the side length of the triangle in the TEM micrograph as the SFT size, the size distribution of SFTs produced by different ion irradiation values is shown in Figure 8a. Here, only the SFTs whose sizes are larger than 2 nm are taken into account. The size distribution of produced SFTs varies under different irradiation conditions. It is concluded that the most probable distribution in size of SFTs is increased from 2.5 nm to 4 nm when increasing the nuclear stopping power of incident ions according to Table 1, and the average size of all the statistic SFTs is positively related to the nuclear stopping power (in Figure 8b). For the higher nuclear stopping power, a heavy ion impact can deposit more energy in the displacement collision cascade, which may result in the larger SFTs.

## 3. Materials and Methods

Polycarbonate membrane (Makrofol, Leverkusen, Germany) of a 30 μm thickness was irradiated at the Heavy Ion Research Facility in Lanzhou (HIRFL), with 9.5 MeV/u ^209^Bi^31+^ ions at a normal incidence with a fluence of 1 × 10^9^ ions cm^−2^. According to the Monte Carlo simulations using SRIM-2013, the electron energy loss and projected range of 9.5 MeV/u ^209^Bi^31+^ in PC are 14 keV/nm and 146 μm, respectively. The calculations indicate that an energetic ion is sufficient to generate a homogeneous and continuous latent ion track in a PC membrane [28]. In order to produce highly cylindrical nanopores by etching in the PC membrane, each side of the membrane was exposed to ultraviolet light for 2 h to enhance the track etching rate [29]. The membrane was chemically etched in 5 moL/L NaOH solution at 50 °C to form cylindrical nanopores whose diameter can be controlled by adjusting the etching time. During the etching process, an ultrasonic field was applied to achieve homogeneous etching. Subsequently, a thin Au layer with a thickness of 50 nm was sputtered onto one side of the prepared template, which was reinforced by a 20 μm Cu film that was electrochemically deposited on it to form an Au/Cu substrate. This Au/Cu substrate acted as the cathode, while a platinum electrode was used as the anode. A total of 0.1 moL/L Na_3_Au(SO_3_)_2_ electrolyte was employed to grow the gold nanowires in a porous template using the electrochemical deposition technique. Gold NWs with different diameters were prepared by using the electrochemical deposition method with a 1.2 V electrodepositing voltage at 50 °C. More details about the fabrication of various nanowires using this technique in our group has been published elsewhere [19]. In this work, we have prepared gold nanowires with diameters in the range of 20–90 nm and a length of up to 30 μm.

In order to characterize the gold nanowires, the PC template was dissolved by using dichloromethane (CH_2_Cl_2_). In the next step, the nanowires were cleaned three times with fresh dichloromethane, to remove the residual PC material on the nanowire surface. After that, gold nanowires were peeled off from the Au/Cu substrate in dichloromethane solution ultrasonically, and were homogeneously dispersed into dichloromethane solution. Subsequently, the solution droplets were transferred to a TEM grid with a porous carbon membrane, and the gold nanowires left the porous membrane after the dichloromethane solution was evaporated completely. The irradiation experiment was carried out using a 320 kV platform at the Institute of Modern Physics, Chinese Academy of Sciences (IMP, CAS), while keeping the gold nanowires dispersed on the TEM grid. The nanowires were irradiated in a vacuum chamber at room temperature in the mode of beam direction perpendicular to the TEM grid. The detailed irradiation parameters in the experiment are presented in Table 1. The electronic stopping power, nuclear stopping power, and ion projecting range in gold were calculated by using SRIM-2013 software (J. F. Ziegler, Annapolis, MD, USA). After irradiation, the surface modification, sputtering particles, and structural changes in gold nanowires were studied by high resolution TEM (FEI, Hillsboro, OR, USA) and SEM (FEI, Hillsboro, OR, USA).

## 4. Conclusions

Gold nanowires with a single crystalline structure were electrochemically prepared in etched ion track templates and irradiated by MeV heavy ions to investigate the interaction between the heavy ion and the one-dimensional materials. It was found that the surface of the gold nanowires was strongly modified by the ion irradiation and the modification effect was increased with a decreasing nanowire diameter. After the ion irradiation, craters with and without a protrusion on the gold nanowires were observed with different shapes, and these were related to the deposited energy in the displacement cascade and the spike position. The deposited energy in the displacement cascade may influence the crater size, and the spike position probably led to different crater formation mechanisms and crater shapes. Here, plastic flow and micro-explosion were considered to be the basic mechanisms for the formation of large craters. An enhanced sputtering effect was also observed and it was confirmed that the number of sputtered nanoparticles increases with an increasing nanowire diameter when under the same ion irradiation conditions. Besides the surface modification, the structural changes inside the gold nanowires were also monitored after the ion irradiation, regarding the formation of SFTs. In this work, three types of SFTs were observed, i.e., perfect SFTs, truncated SFTs, and a group of intermixed SFTs. By analysing the size distribution of these SFTs, produced by different ions, it was concluded that the most probable distribution for the size of the SFTs was increased from 2.5 nm to 4 nm with the increase of the nuclear stopping power.

## Figures and Tables

**Table 1:** Parameter settings in irradiation experiment.

Parameter Settings			Stopping Power in Gold		
Ion Species	Energy (MeV)	Fluence (Ions cm^−2^)	${\left(\frac{dE}{dx}\right)}_{e}$ (keV/nm)	${\left(\frac{dE}{dx}\right)}_{n}$ (keV/nm)	Projected Range (nm)
Xe	1.4	1 × 10^14^	1.44	4.83	143
Xe	4		3.21	3.33	441
Kr	1.4		1.28	2.15	237
Kr	3		2.51	1.54	532

## Supplementary Materials

The following are available online at http://www.mdpi.com/2079-4991/7/5/108/s1.
